# Clinical malaria case definition and malaria attributable fraction in the highlands of western Kenya

**DOI:** 10.1186/1475-2875-13-405

**Published:** 2014-10-15

**Authors:** Yaw A Afrane, Guofa Zhou, Andrew K Githeko, Guiyun Yan

**Affiliations:** Climate and Human Health Research Unit, Centre for Global Health Research, Kenya Medical Research Institute, Kisumu, Kenya; School of Health Sciences, Jaramogi Oginga Odinga University of Science and Technology, Bondo, Kenya; Program in Public Health, College of Health Sciences, University of California, Irvine, CA USA

**Keywords:** Clinical malaria case definition, Attributable fraction of fevers to malaria, Highlands, Kenya

## Abstract

**Background:**

In African highland areas where endemicity of malaria varies greatly according to altitude and topography, parasitaemia accompanied by fever may not be sufficient to define an episode of clinical malaria in endemic areas. To evaluate the effectiveness of malaria interventions, age-specific case definitions of clinical malaria needs to be determined. Cases of clinical malaria through active case surveillance were quantified in a highland area in Kenya and defined clinical malaria for different age groups.

**Methods:**

A cohort of over 1,800 participants from all age groups was selected randomly from over 350 houses in 10 villages stratified by topography and followed for two-and-a-half years. Participants were visited every two weeks and screened for clinical malaria, defined as an individual with malaria-related symptoms (fever [axillary temperature ≥ 37.5°C], chills, severe malaise, headache or vomiting) at the time of examination or 1–2 days prior to the examination in the presence of a *Plasmodium falciparum* positive blood smear. Individuals in the same cohort were screened for asymptomatic malaria infection during the low and high malaria transmission seasons. Parasite densities and temperature were used to define clinical malaria by age in the population. The proportion of fevers attributable to malaria was calculated using logistic regression models.

**Results:**

Incidence of clinical malaria was highest in valley bottom population (5.0% cases per 1,000 population per year) compared to mid-hill (2.2% cases per 1,000 population per year) and up-hill (1.1% cases per 1,000 population per year) populations. The optimum cut-off parasite densities through the determination of the sensitivity and specificity showed that in children less than five years of age, 500 parasites per μl of blood could be used to define the malaria attributable fever cases for this age group. In children between the ages of 5–14, a parasite density of 1,000 parasites per μl of blood could be used to define the malaria attributable fever cases. For individuals older than 14 years, the cut-off parasite density was 3,000 parasites per μl of blood.

**Conclusion:**

Clinical malaria case definitions are affected by age and endemicity, which needs to be taken into consideration during evaluation of interventions.

## Background

Evaluation of malaria interventions, such as long-lasting impregnated nets (LLINs), indoor residual spraying (IRS), drugs and vaccines trials depends on clinical definition of the disease, which is still a challenge due to lack of distinct malaria specific clinical features. Malaria case definitions for interventions differ from those used in clinical care in that high specificity is needed and this is complicated by the relatively high prevalence of asymptomatic parasitaemia in endemic areas [1]. Parasite density thresholds are used in definition of clinical malaria in evaluation of interventions to make the definition more specific. Defining a cut-off level is complicated by the fact that the pyrogenic threshold of parasite density varies both with age and differing levels of immunity [2–5]. Immunity is linked to transmission intensity and this varies widely in many parts of Africa [3], and variations over relatively short distances can still influence clinical definitions [4].

The clinical signs of malaria are nonspecific and parasitaemia accompanied by clinical symptoms consistent with malaria does not necessarily imply clinical malaria especially in endemic areas [4]. To measure morbidity in endemic areas, a clear case definition of clinical malaria is needed [5]. Whereas in non-endemic areas, peripheral parasitaemia accompanied by fever could be used to define clinical malaria, in endemic areas this is not so since over 60% of individuals could always have asymptomatic parasitaemia. Illnesses such as typhoid fever, which has an accompanying fever, could be confused with clinical malaria because of accompanying parasitaemia. In the African highlands, malaria transmission could vary so much depending on the altitude and topography. There is, therefore, a clear need for locally appropriate definitions for malaria in these sites.

Clear case definitions of malaria are an essential means of evaluating the effectiveness of present and proposed interventions in malaria. Several studies have estimated parasite cut-off densities in different settings, using the point at which the sensitivity and specificities are both highest as the optimum case definition. This definition is most appropriate for clinical trials for which higher specificity is more important than that for clinical diagnosis and treatment, where high sensitivity is paramount. Definitions range from any to 7,000 parasites per microlitre (μl) of blood depending on age [6, 7], season [8] and level of endemicity [9, 10]. The fraction of fevers attributable to parasitaemia can also be used to calculate the number of fevers that would be eliminated if malaria was eradicated [6, 7]. Logistic regression is used to model the risk of fever as a continuous function of the parasite density [11].

The distribution pattern of febrile malaria may be used to infer environmental risk factors and thus providing crucial information for the development of new prevention or intervention methods. The topography in the highlands of western Kenya is made up of hills and valleys and this affects the epidemiology of malaria [9, 10, 12]. Some valleys are U-shaped, where drainage of rain water or streams are slow and, therefore, create breeding sites in the forms of swamps for mosquitoes. Others are V-shaped with terrain for fast flowing water which does not support mosquito breeding [10, 13]. Studies have from Himeidan *et al.* [14] showed that mosquitoes breed in the valleys with very little or no breeding sites in the hills depending on the time of the year.

In this study, Clinical malaria cases were quantified through active case surveillance and asymptomatic malaria infection screening and used the data to define age-specific clinical cases in highland areas in western Kenya. This was part of a study to test different combinations of malaria interventions in the area.

## Methods

### Study site

The study was conducted in a highland site in Iguhu, Kakamega south district, western Kenya. Ten clusters of villages were selected which were stratified by topography. Individual households are spread almost everywhere from the bottom of the valleys (valley bottom), through the middle of the hills (mid-hill) up to the top of the hills (hill top). The Yala River transects the site and most of the mosquito breeding takes place in cultivated swamps in the valley and also at the edges of the several streams in the site, therefore malaria transmission intensity varied from valley bottom (with the highest parasite prevalence rate) to hill tops (with lowest parasite prevalence rate) [12]. Malaria transmission in these sites is seasonal and peaks in June and July, two months after the onset of the long rainy season [15] The hot and dry season is from January to March and this marks the lowest malaria transmission season; changes in transmission is minimal during the short rainy season from October to November [16]. *Plasmodium falciparum* is the predominant malaria species in the study sites and it is transmitted by *Anopheles gambiae, Anopheles arabiensis* and *Anopheles funestus* [10, 12, 16, 17].

### Active case surveillance

A cohort of over 1,800 participants of all ages was selected randomly from over 350 houses in the 10 villages in Iguhu for the longitudinal study. All houses in the study sites had been mapped and assigned a unique identification number which has been previously described [18]. Participating households were randomly selected from these identified houses. Participants were visited every two weeks and screened for clinical malaria. A clinical malaria case was defined as an individual with malaria-related symptoms (fever [axillary temperature ≥ 37.5°C], chills, severe malaise, headache or vomiting) at the time of examination or 1–2 days prior to the examination in the presence of a *P. falciparum* positive blood smear [19]. During each visit, our team, which consisted of a lead laboratory technician, and a community health worker, talked to the matriarch who has information on the health of everyone in the house, to find out about residents who may be having or have experienced fever within the last 48 hours or who suspected they have malaria. Blood smears were taken to prepare a thin and thick smear on a labeled slide from each participant who had fever or perceived to have malaria. Body temperature, taken with a digital thermometer and the symptoms and the signs of the illness were recorded. Clinical cases were referred to the nearest hospital for free treatment. Each participant was given a unique identification number that corresponds to the household and their village. Each participant was also given an identity card used to obtain free treatment for malaria at the hospital anytime they had fever or perceived they had malaria. Records of immigration, deaths and births were made at each visit. Date of birth, gender, malaria prevention practices of each person was recorded for each participant. The cohort was followed from March 2007 - July 2009.

### Malaria asymptomatic infection cross-sectional screening

During the dry (February and March) and rainy (June and July) seasons, which represent the low and high malaria transmission seasons in the area respectively, the cohort population was screened for asymptomatic malaria infection. Thick and thin blood smears were made from each individual. Temperature, symptoms of malaria, if any, were recorded. Four surveys were done, two each in 2008 and 2009. Blood smears were not analysed immediately and, therefore, participants who had positive parasitaemia had no treatment. Nevertheless, each participant already had an ID card to access free medical care in the health facilities.

### Laboratory slide readings

The thin and thick blood smears were air-dried. The thin smears were fixed in methanol and stained in 4% Giemsa for 30 minutes. Two experienced laboratory technicians who were blinded to each other’s results examined the slides under × 1,000 oil immersion to obtain a species specific parasite count. Results were later compared and if there were any discrepancies in slide readings, a third and more experienced technician was brought in to confirm the diagnoses. Parasite density was scored against 200 leukocytes when the slide was positive; otherwise, the whole slide was carefully scanned before being declared negative. Parasite densities were converted to number of parasites per microliter of blood, assuming a leukocyte count of 8,000 cells/μL [15]. For quality control, 10% of the blood smears were randomly selected and read by highly an experienced microscopist.

### Informed consent and ethical clearance

Ethical clearance was given by the Ethical Review Committee (ERC) of the Kenya Medical Research Institute, Kenya and Institutional Review Board (IRB) of University of California, Irvine, USA. Written consent was obtained from all participants. Written consent for children (<18 years of age) was provided by the participants and their parents or guardians. Inclusion criteria were: provision of informed consent, age >6 months at recruitment, and no reported chronic or acute illness except malaria. Exclusion criteria were: those who were unwilling to participate in the study, those who planned to move out of the study area and those with no reported chronic or acute illness except malaria.

### Data analysis

Data were entered into Microsoft Excel spreadsheet and processed and analyzed using SPSS software package (SPSS 15.0 for Windows, Chicago, IL) and JMP Statistical software [20]. Sampled households were divided into three topographical groups according to the house location, i.e., valley bottom, mid-hill, and hill top [21]. Individuals who lived in the same topographical area were pooled together to calculate clinical malaria incidence rate and prevalence of asymptomatic malaria infections. Incidence rate of clinical malaria through active surveillance was calculated as cases per 1,000 people per year. The difference in the incidence rate of clinical malaria in different topographical areas was compared using a paired *t*-test with Bonferroni correction. Asymptomatic malaria infection rate was calculated as ratio of infected individuals over total samples. The differences in incidence rate of clinical malaria and asymptomatic infection rates between the topographical sites were determined by chi-square test, between season and year differences were tested the same way.

### Malaria case definitions and attributable fraction

The case definitions were derived using logistic regression methods. The logistic regression model used was [22]


where *P* is the probability that a participant with parasites at a density *x* had a fever, and where _τ_ is the power function of the parasite density. The power function was derived using maximum likelihood estimation for the different age groups and was used to model the relationship between fever and parasite density as a continuous function. The cutoff parasite density for different study area and age groups were determined by the intersection of sensitivity and specificity curves. The malaria attributable fraction (MAF) was calculated as the reduction in incidence that would be observed if the population were entirely unexposed, compared with its current (actual) exposure pattern.

## Results

### Incidence of clinical malaria through active case surveillance

**Figure 1:**
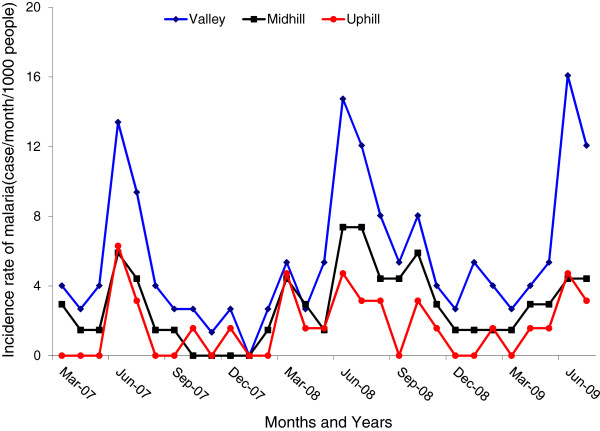
**Incidence rate of clinical malaria through active case surveillance in the valley bottom, midhill and uphill populations.**

Incidence rate of clinical malaria among the study participants detected through active case surveillance was found to be associated with topography of the area. It was clustered around the valley bottoms. The incidence rate of clinical malaria was highest in the population living in the valley bottoms (5.0% cases per 1,000 population per year) compared to the population in the mid-hill (2.2% cases per 1,000 population per year) (t = 9.90, df =57, P < 0.0001) and in the up-hill population (1.1% cases per 1,000 population per year) (t = 5.37, df =57, P < 0.0001). The population in the mid-hill sites had a higher incidence of clinical malaria than the uphill residents (t = -11.6, df =57, P < 0.0001; Table 1). Incidence rate of clinical malaria was higher during the main rainy season compared to the dry season. There was an almost three-fold increase in clinical malaria prevalence during the rainy season compared with the dry season (Figure 1). Fever cases were found to be consistently higher than confirmed clinical malaria cases in all topographical sites and across all age groups (Table 1).

**Table 1:** **Malaria infection levels of the cohort in western Kenya**

Surveillance	Valley bottom	Midhill	Uphill
Population	646	598	605
Clinical malaria incidence rate	5.0a	2.2b	1.1c
Asymptomatic infection (%)	10.78a	4.49b	1.85c
Gametocytes	1.04a	0.4b	0.19c

Letters following the numerical values indicate the results of multiple comparison tests, and values with the same letter were not statistically significant at *P* _ 0.05 within a season.

### Asymptomatic malaria parasite infections

Individuals living in the valley bottoms had the highest asymptomatic infection (10.78%) followed by those living in the mid-hill (4.49%) and up-hill (1.85%) sites (F = 6.78, df = 2,17, P = 0.07). Infections at all sites were also higher during the rainy season (compared to the dry season 8.8% vs 4.09%; F = 4.23, df = 2, 18, P = 0.05). *Plasmodium falciparum* was the only malaria parasite encountered. Gametocytes decreased with increase in elevation but the differences between them was not significant (1.04 vs. 0.40 vs 0.19; F = 0.69, df = 2,17, P = 5.12; Table 1).

### Malaria parasite case definitions

**Figure 2:**
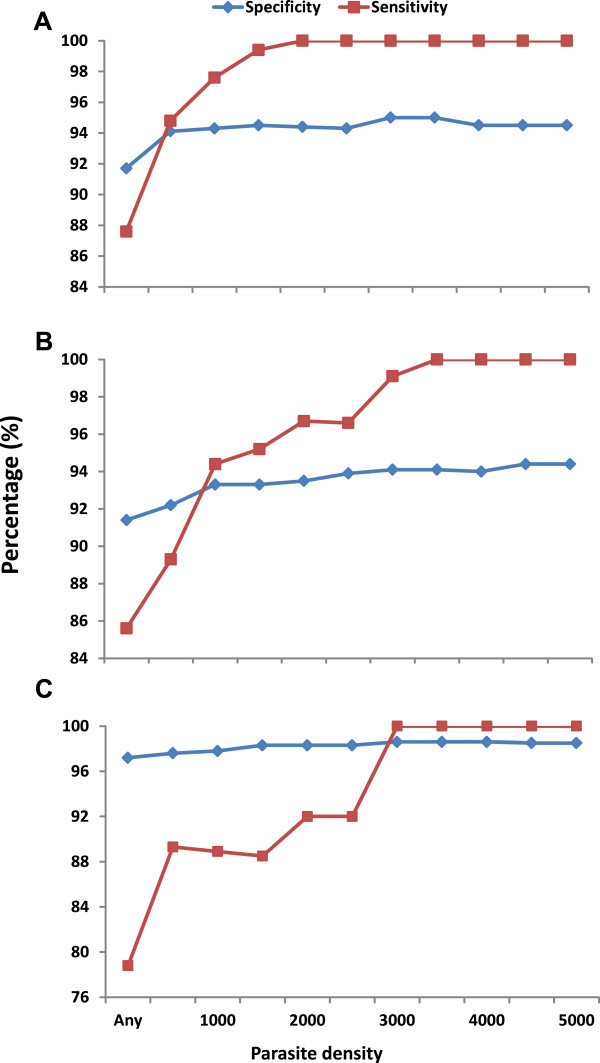
**Sensitivity and specificity values at given parasite density for different age groups (A) for children age less than 5, (B) Children between 5–14 years of age and (C) for people older than 15 years.**

The optimum cut-off parasite densities through the determination of the sensitivity and specificity showed that in children less than five years of age, 500 parasites per μl of blood could be used to define the malaria attributable fever cases for this age group. In children between the ages of 5–14, a parasite density of 1,000 parasites per μl of blood could be used to define the malaria attributable fever cases. For individuals older than 14 years, the cut-off parasite density was higher. The cut-off value to determine the malaria attributable fever was found to be 3,000 parasites per μl of blood. Figure 2 shows the sensitivity and specificities of various cut-off parasite densities used in the definition of clinical malaria in the various age groups.

### Malaria attributable fractions (MAFs)

From the model, the mean malaria-attributable fraction or the probability that any individual fever case was attributable to malaria was 0.65 in children less than five years, 0.58 in the age group 5–14 years and was 0.32 in the age group of ≥ 15 years. As expected, the adult group had significantly lower malaria-attributed fraction among the three age groups (Table 2), this is very likely due to the immunity developed in this age group, i.e., adults did tolerate some level of parasitemia without developing the fever.

**Table 2:** **Malaria case definition parasite density cut-offs and fever attributable fraction of the cohort in western Kenya**

Surveillance	<5	5-14	>14
Population	765	691	861
Cut-off values (parasites per μl of blood)	500	1000	3000
Malaria attributable fraction	0.65	0.58	0.32

## Discussion

This study quantified clinical malaria cases through active case surveillance and asymptomatic screening for malaria infections. Fever and parasite densities were used to define age-specific clinical malaria cases in a highland area in western Kenya. There was clustering of clinical malaria cases and asymptomatic malaria infections around the bottom of the valley both through active case surveillance and the screening of the cohort for asymptomatic infections respectively and with very clear seasonality. Individuals 15 years and older had higher cutoff points of parasite density used in defining clinical malaria than those younger than that age. From the malaria attributable fraction estimations, many of the fevers in the population could be attributed to clinical malaria.

Malaria transmission in most parts of the highlands of East Africa and indeed in western Kenya is spatially and temporally driven by climate variability and topography [15, 16, 23, 24]. The topography of the highlands comprises hills, valleys, and plateaus. Rivers and streams run along the valley bottoms in the valley ecosystem and swamps are a common feature which creates breeding sites for mosquitoes [9, 17]. Unlike in lowland plains where drainage is poor and mosquito breeding habitats have an extensive distribution, the majority of breeding habitats in the hilly highlands are confined to the valley bottoms because the hillside gradients provide efficient drainage [25]. The non-homogeneous distribution of larval breeding habitats affect adult vector spatial distribution, and consequently may lead to focal malaria transmission [12, 15]. This clearly explains the clustering of clinical cases and asymptomatic infections around the valley bottoms. Recognizing transmission hotspots would permit control efforts to be directed at specific geographic areas, and thus reducing costs and increasing effectiveness. Control of transmission in such “hotspots” might also eventually lead to reduction of case numbers in “cool spots”.

The results reveal an increasing trend in the incidence of clinical malaria in the area. Malaria infections increased each year during the study period. These results supports what other studies have reported in western Kenya [16, 26] on the resurgence of clinical malaria infection after initial decline in 2006. More efforts are needed from the malaria control programme to tackle the increasing malaria in the area. Previous studies, such as Githeko *et al.* [12], supports the results of our study on the clustering of malaria infections in valley bottoms.

The differences in cut off values for parasite density with increase in age could be attributed to the fact that older people could tolerate higher densities of parasites than younger ones, due in part to immunity build up. The cut-off value for parasites densities to define clinical malaria for individuals older than 15 years was 3,000 parasite per μl of blood showing how much they could tolerate malaria parasite in their bodies without getting sick. In children under five years of age, they only require 500 parasites per μl of blood and children between 5–14 years require 1,000 parasites per μl of blood. These underlie how immunity build up affects asymptomatic parasite carriage. Immunity to malaria infection depends on endemicity of the disease in the area. Thus, people living in the valley bottoms of our study area may have more immunity to malaria than the mid-hill and uphill population. Endemicity of malaria could also be a factor in determining cut-off values for parasite density in defining clinical malaria [27, 28]. In the present study, clinical malaria was not defined according to transmission levels even though we found that incidence of malaria to be different by topography and many published studies supports this fact.

In this area, over 60% of participants with fever or a history of fever were not parasitaemic, underlying the fact that fever is not a specific marker for clinical malaria. It was found that for people above the cut-off value for the definition of clinical malaria, over 90% of the fevers could be attributed to malaria. These results show the high specificity of our results and the fact that fever alone with parasitaemia cannot be used to define clinical malaria. One limitation to this study was that fever cases might have been missed in between home visits that might not have been added to the overall fever cases.

It can be concluded that in highland areas where malaria transmission varies by topography, clinical malaria should be defined by age and also by endemicity when evaluations of interventions are being conducted. However, the situation where intervention groups and control groups are being evaluated will bring challenges because whereas the control groups might have higher parasitaemia, the intervention groups might have lower parasitaemia which might bring distortions in the definition for clinical malaria even in the same age group or transmission settings.
